# Clinical significance of LINC01158 in breast cancer and inhibition of proliferation and metastasis of breast cancer cells by regulating miR-711

**DOI:** 10.1186/s41065-025-00452-1

**Published:** 2025-05-22

**Authors:** Xiaohe Lan, Zibai Guo, Linmei Lin, Wanqi Lin, Yi Zheng, Yabing Liu

**Affiliations:** 1 Guangzhou University of Chinese Medicine, Guangzhou, 510006 Guangdong China; 2 Department of Traditional Chinese Medicine, Guangdong Provincial People’s Hospital (Guangdong Academy of Medical Sciences), Southern Medical University, Guangzhou, 510080 Guangdong China; 3 Breast Center I, Zhongshan City People’s Hospital, Zhongshan, 528400 China; 4 Blood Transfusion Department, The First Hospital of Putian City, Putian, 351100 China; 5 Fujian Health College, Fuzhou, 350101 China; 6 Putian University, Putian, 351100 China; 7 Department of Oncology, Yueyang People’s Hospital, Yueyang Hospital Affiliated to Hunan Normal University, No.263, Baling East Road, Yueyanglou District, Yueyang City, 414000 Hunan Province China

**Keywords:** Breast cancer, Prognostic value, LINC01158, miR-711

## Abstract

**Objective:**

Breast cancer (BC) has a poor prognosis due to metastasis and recurrence. LINC01158 is aberrantly expressed in breast cancer. Therefore, we investigated the regulatory mechanism and prognostic value of LINC01158 in BC.

**Methods:**

121 patients with BC were enrolled. LINC01158 and miR-711 levels were analyzed by RT-qPCR. Independent predictors of poor BC prognosis were analyzed by multifactorial Cox regression. Kaplan-Meier curves were used to analyze the 5-year survival rate of BC patients. DLR assay verified the relationship between LINC01158 and miR-711 target binding. CCK-8 was used to detect the proliferative capacity of cells. Transwell was used to analyze cell migration and invasion ability.

**Results:**

In BC tissues and cell lines, LINC01158 expression was reduced and miR-711 levels were elevated. Low expression of LINC01158 resulted in a shortened overall survival of BC patients. LINC01158 binds to the miR-711 target and negatively correlates with the level of miR-711. Overexpression of LINC01158 decreased miR-711 levels and reduced BC cell proliferation, migration and invasion. In addition, Cox regression results showed that LINC01158 was an independent prognostic factor for BC.

**Conclusion:**

LINC01158 may be a prognostic marker for BC. Increasing the expression level of LINC01158 could reduce the expression of miR-711, which could inhibit cell proliferation, migration and invasive behaviors, and has the potential to delay the progression of BC.

## Introduction

Breast cancer (BC) is a malignant tumor that originates from the epithelial cells of the breast and is often found in the female population [1]. Early symptoms of breast cancer are not obvious, mainly including breast swelling, breast lumps, enlarged axillary lymph nodes, and other symptoms [2]. Most patients find it difficult to detect it in time, and when obvious symptoms such as breast skin depression and nipple abnormalities appear, the best time for treatment is often missed. In recent years, the development of screening methods has greatly facilitated the early detection and treatment of BC [3], but the prognosis of BC is still unsatisfactory due to its drug resistance and the metastatic and recurrent nature of the tumor [4].

In recent years, more and more researchers have begun to focus on the value of long non-coding RNAs (lncRNAs) and microRNAs (miRNAs) in the prognosis of malignant tumors [5–8]. LncRNAs are associated with the development of malignant tumors and have great potential to become biomarkers for tumor diagnosis, prognosis, and even targeted gene therapy [9]. Moreover, given the important role of lncRNAs in the regulation of cell differentiation and cell cycle [10, 11], they represent a promising and viable source of biomarkers [12]. They could make an important contribution to the diagnosis of BC status, the identification of drug targets, and the assessment of the efficacy of therapeutic interventions [13].

Existing studies have shown that LINC01158 is aberrantly expressed in some cancers, such as glioma [14] and squamous cell carcinoma of the lung [15], and has the potential to be a prognostic marker. LINC01158 was reported to be down-regulated in BC in a study by Jia et al. [16], but the clinical significance and molecular mechanisms were unknown. Furthermore, the presence of a binding site for LINC01158 with miR-711 was predicted through the database and miR-711 was reported to be significantly upregulated in BC [17]. However, the participation of LINC01158 in the progression in BC by targeting miR-711 needs to be further investigated. Therefore, in this study, LINC01158 and miR-711 were used to investigate their prognostic value in BC and their roles in the developmental process of BC.

## Materials and methods

### Patient inclusion

121 BC patients who attended The First Hospital of Putian City from November 2017 to May 2019 were included. Inclusion criteria: (a) all met the relevant diagnostic criteria of BC; (b) all were first-ever, primary BC patients who met the indications for surgery; (c) no treatment such as radiotherapy before surgery. Exclusion criteria: (a) those with coagulation disorders; (b) those with liver and kidney failure; (c) those with other malignant tumors. All patients were treated with surgical resection and the patient’s tumor tissues and paracancerous normal tissues (confirmed by pathological examination) were collected. All patients were followed up for 5 years after surgery.

The research followed the tenets of the Declaration of Helsinki and was approved by the Ethics Committee of The First Hospital of Putian City. In addition, all participants gave informed consent.

### Cell culture and transfected

Three types of breast cancer cells (SKBR3, HS578T, T47D) and human normal mammary epithelial cells MCF-10 A (Shanghai, China) were selected for in vitro experiments. MCF-10 A was cultured in DMEM/F12 (1:1) medium, and SKBR3, HS578T, and T47D were cultured in DMEM medium. The medium contained 10% fetal bovine serum, 10 mg/mL streptomycin, and 100 U/mL penicillin. The incubation temperature was 37 ℃ and the CO_2_ concentration was 5%.

LINC01158 overexpression plasmid (oe-LINC01158) and empty vector (oe-NC), miRNA blank control (miR NC), and miR-711 mimic (miR mimic) were purchased from RiboBio (Guangzhou, China). SKBR3 and T47D cells were transfected according to the steps of the Lipofectamine 3000 kit (Invitrogen, USA).

### RNA extraction and real-time quantitative reverse transcription PCR (RT-qPCR)

Frozen cancer tissues and normal tissue samples adjacent to the cancer were thoroughly crushed. Total RNA was extracted by adding 1 mL of Trizol reagent to the crushed tissues. Subsequently, cDNA was extracted by a reverse transcription kit (Takara, Dalian, China). PCR was performed according to the instructions of the KAPA SYBR Rapid One-Step Kit (Roche, Switzerland), using GAPDH and U6 as endogenous references for LINC01158 and miR-711. Results were normalized to Ct values using the 2^−ΔΔCT^ method.

### Cell proliferation assay

Cell proliferative capacity was determined using CCK-8. 200 µL of cell suspension was inoculated into 96-well plates with three composite wells per group. Cells were cultured for 24 h, 48 h, and 72 h, respectively. 10 µL of CCK-8 solution was added, and the cells were incubated for 1 h. The cells were removed and shaken for 30 s on an enzyme marker to measure OD 450 nm.

### Cell migration and invasion assay

A 24-well Transwell chamber or chamber coated with 50 mg/L Matrigel was used to assess cell migration and invasion. Transfected cells (2 × 10^4^ cells/well) cultured with 100 mL DMEM basal medium (without FBS) were placed in the upper chamber of the Transwell. DMEM complete medium with FBS was added to the lower chamber. After 24 h of incubation, migrated or invaded cells were fixed with methanol (10 min) and stained with crystal violet for 20 min. The excess dye was then washed away and five randomly selected fields of view were observed, photographed, and counted using a light microscope equipped with a camera.

### Dual-luciferase reporter assay

Gene plasmids were designed based on bioinformatics analyses of both binding sites. Wild-type LINC01158 (WT- LINC01158) and mutant LINC01158 (MUT- LINC01158) recombinant plasmids were constructed. The recombinant plasmids were co-transfected into cells with miR-711 mimic and miR-711 inhibitor using Lipofectamine 3000 transfection reagent. 48 h after transfection, luciferase activity was measured using the Dual-Luciferase Kit (Beyotime, Shanghai).

### Bioinformatics analysis

Prediction of LINC01158 and miR-711 target binding sites using the lncRNASNP2 human database.

### Statistical analysis

All experiments were repeated at least three times in parallel and the results were expressed as mean ± standard deviation. Cox analysis and patients’ clinical physiological data were analyzed and tabulated using SPSS, and the rest of the data were analyzed and plotted using GraphPad. Differences between groups were analyzed using t-tests. The prognosis of BC patients was analyzed using Kaplan-Meier plotter curves. A *p*-value of less than 0.05 indicates that the difference is statistically significant.

## Results

### Expression of LncRNA LINC01158

**Fig. 1:**
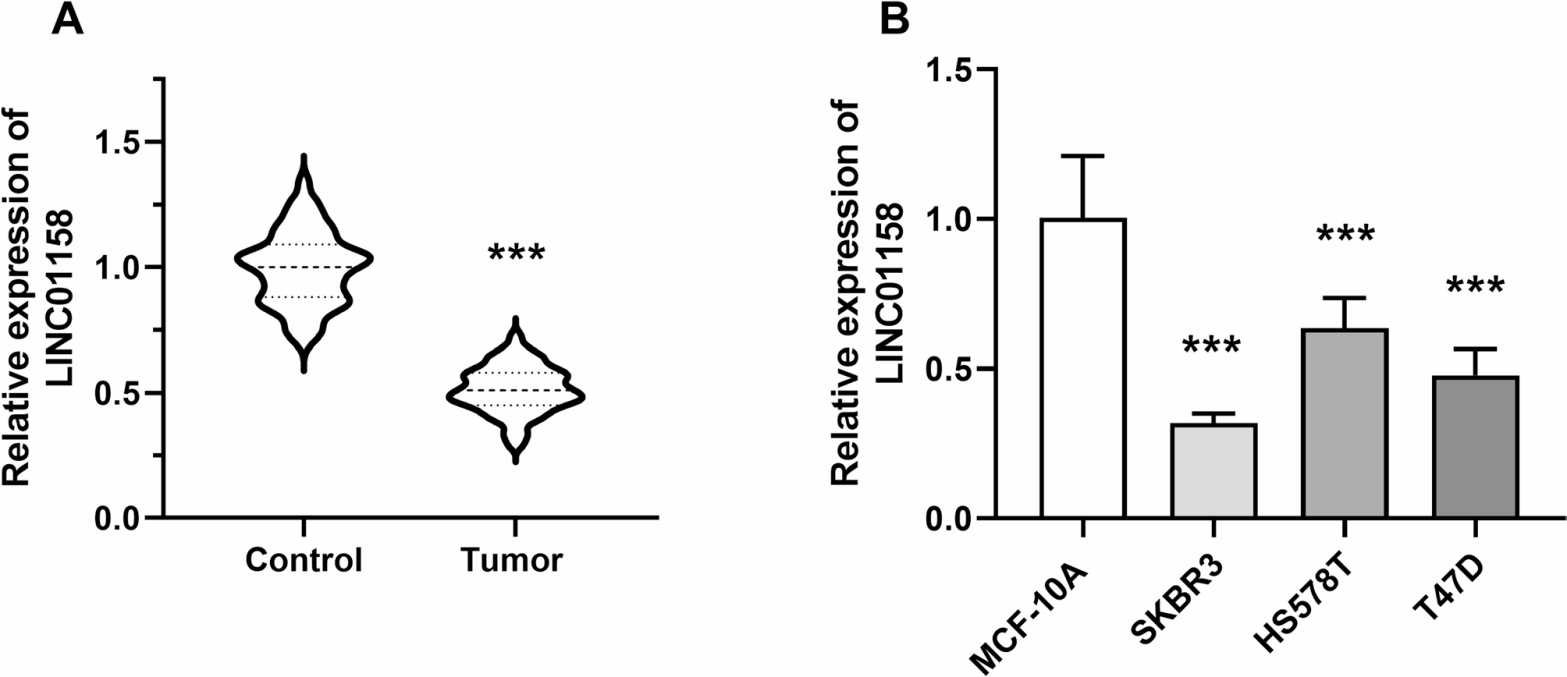
LINC01158 expression in BC. **A** LINC01158 level in BC tissues was lower than in normal tissues; **B** LINC01158 level in BC cell lines was lower than in MCF-10 A (*** *P* < 0.001 vs. control or MCF-10 A)

LINC01158 levels were lower in cancerous tissues of BC patients compared to normal tissues (*P* < 0.001, Fig. 1A). The same result was found for LINC01158 levels in cells. Compared to MCF-10 A cells, the level of LINC01158 was down-regulated in SKBR3, HS578T, and T47D (*P* < 0.001, Fig. 1B). Furthermore, the low expression of LINC01158 in BC cancer tissues was associated with TNM staging and lymph node metastasis (LNM) (*P* < 0.05), independent of other factors such as age, pathological differentiation, and tumor size (*P* > 0.05, Table 1).

**Table 1:** Correlation between LINC01158 levels and clinical features in BC patients

Parameters	Total *n* = 121	TPRG1-AS1 expression levels		*P*
		Low (*n* = 63)	High (*n* = 58)	
Age				
< 50	53	30	23	0.378
≥ 50	68	33	35	
Pathological differentiation				
Low	52	32	20	0.070
Moderate + high	69	31	38	
TNM stage				
I + II	63	25	38	**0.004**
III+IV	58	38	20	
Lymph node metastasis				
NO	75	33	42	**0.023**
YES	46	30	16	
Tumor size (cm)				
≤ 2	65	30	35	0.161
> 2	56	33	23	
Progesterone receptor (PR)				
Positive	55	29	26	0.894
Negative	66	34	32	
Estrogen receptor (ER)				
Positive	61	33	28	0.652
Negative	60	30	30	
Human epidermal growth factor receptor 2 (HER2)				
Positive	63	36	27	0.244
Negative	58	27	31	

### Prognostic value of LINC01158 in patients with BC

**Fig. 2:**
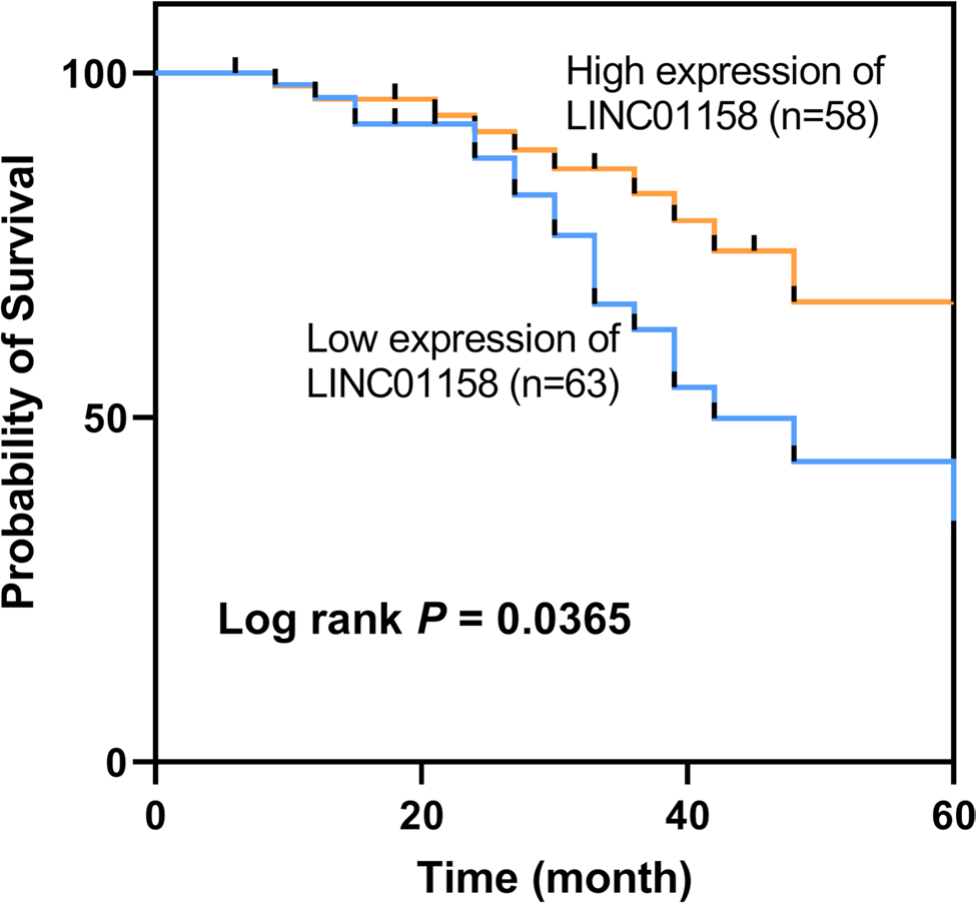
The prognostic role of LINC01158 in BC. Patients in the LINC01158 low expression group had shorter overall survival (Log-rank *P* = 0.0365)

Figure 2 demonstrates that low LINC01158 level was associated with shorter overall survival in BC patients (Log-rank *P* = 0.0365). LINC01158 was also a risk factor for the clinical prognosis of BC patients by multifactorial Cox survival analysis (*P* = 0.016, HR = 2.926). In addition, TNM stage (*P* = 0.022, HR = 0.331) and LNM (*P* = 0.030, HR = 0.424) were also independent prognostic factors for BC patients (Table 2).

**Table 2:** Multivariate Cox analysis of LINC01158 and clinical parameters with overall survival

Characteristics	Multivariate analysis		
	*P*	HR	95%CI
LINC01158	**0.016**	2.926	1.218–7.032
Age	0.309	0.654	0.289–1.482
Pathological differentiation	0.078	0.456	0.190–1.093
TNM stage	**0.022**	0.331	0.129–0.851
Lymph node metastasis	**0.030**	0.424	0.196–0.918
Tumor size	0.096	2.075	0.878–4.904
Progesterone receptor (PR)	0.407	1.387	0.640–3.007
Estrogen receptor (ER)	0.125	0.546	0.252–1.183
Human epidermal growth factor receptor 2 (HER2)	0.822	1.094	0.501–2.389

### Targeted binding of LINC01158 and miR-711

**Fig. 3:**
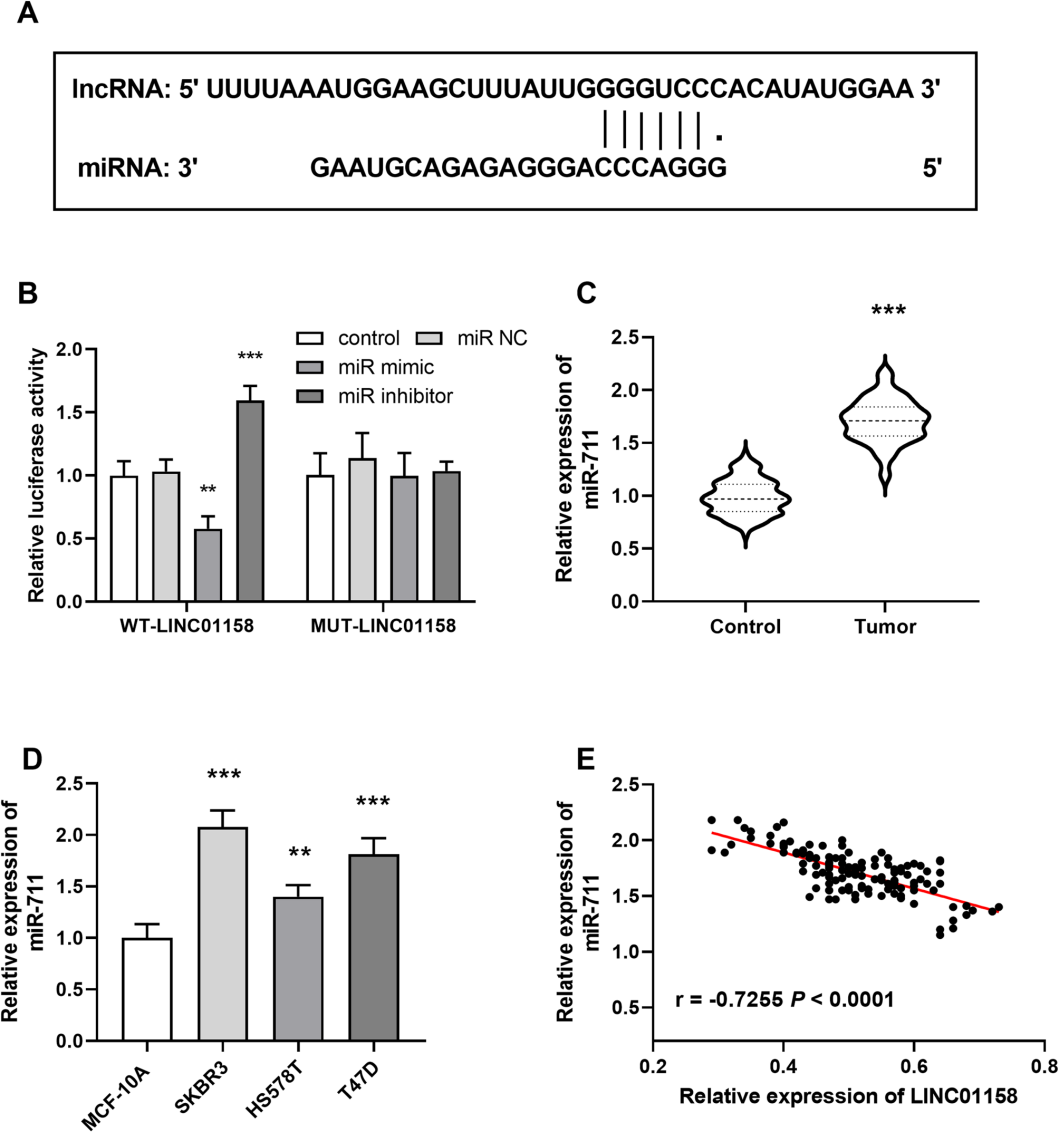
Targeted binding of LINC01158 to miR-711. **A** Predicted target binding location of LINC01158 to miR-711; **B** DLR assay demonstrates targeting between LINC01158 and miR-711; **C** miR-711 expression was increased in BC tissues; **D** miR-711 expression was increased in BC cell lines; **E** miR-711 level was inversely correlated with LINC01158 (*r*=-0.7255, *P* < 0.0001) (*** *P* < 0.001 vs. control or MCF-10 A)

Figure 3A demonstrates the binding sites of LINC01158 and miR-711 in the database. DLR experiments demonstrated a target-binding relationship between the two. miR-711 mimics decreased WT-LINC01158 luciferase activity, whereas miR-711 inhibitors increased the activity (*P* < 0.001), but had no effect on PWRN1-MUT (*P* > 0.05; Fig. 3B). miR-711 illustrated elevated in cancer tissues (*P* < 0.001, Fig. 3C). The same trend was shown in cells, with miR-711 levels significantly elevated in BC cancer cell lines (*P* < 0.01, Fig. 3D). LINC01158 and miR-711 expression were negatively correlated, with increased expression of LINC01158 leading to decreased levels of miR-711 (*r* = -0.7255, *P* < 0.0001; Fig. 3E).

### LINC01158 and miR-711 together affect the physiological function of cells

**Fig. 4:**
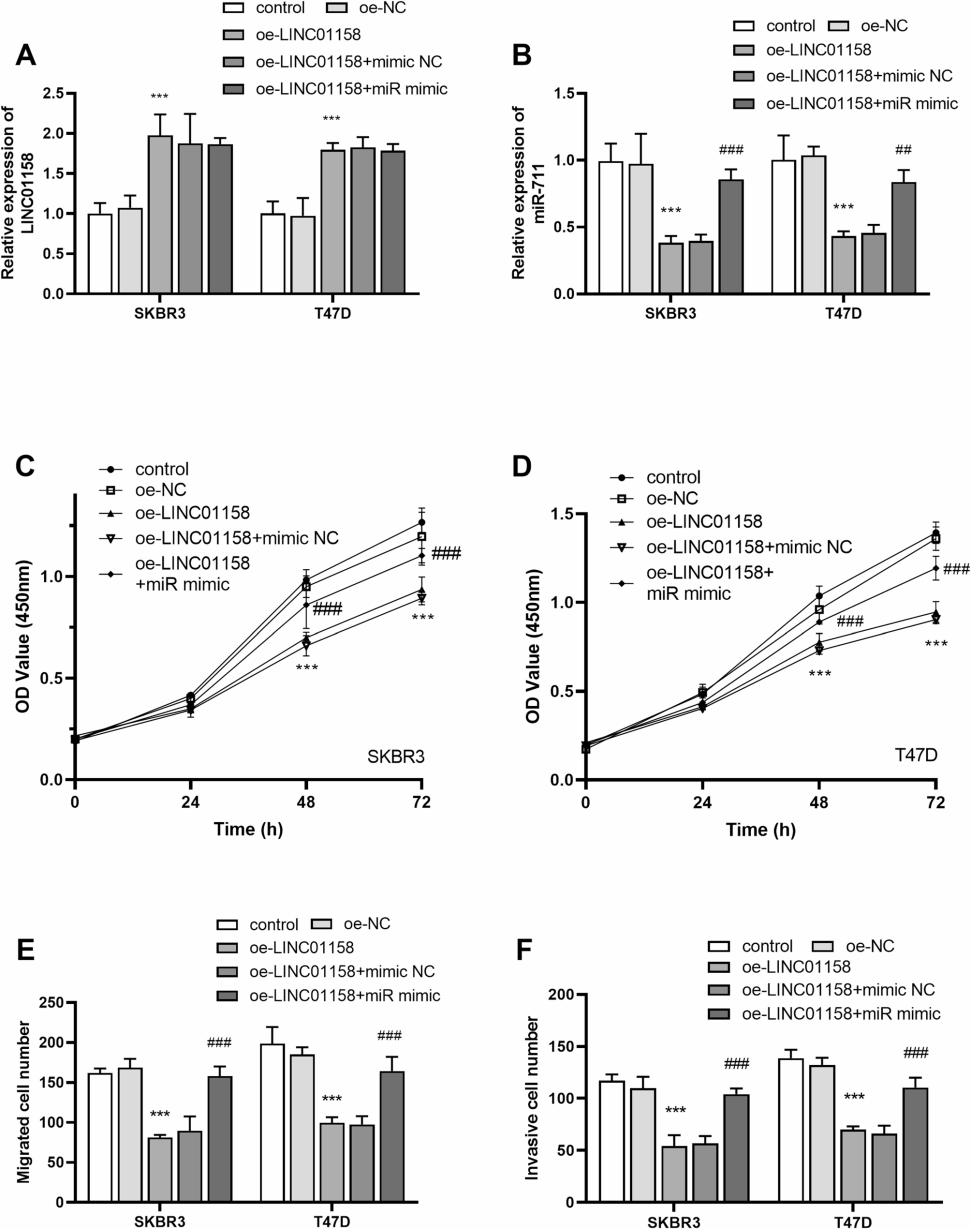
LINC01158 and miR-711 co-regulate cell physiological activity. **A** and **B** Transfection of overexpressed LINC01158 resulted in its level increase and **a** decrease in miR-711 level; transfection of oe-LINC01158 + miR mimic had no effect on LINC01158 level, and miR-711 expression was markedly up-regulated; **C** and **D** LINC01158 overexpression reduced the proliferative capacity, whereas the addition of miR mimic reversed this effect; **E** oe- LINC01158 reduced the migratory capacity of the cells, whereas the addition of miR mimic reversed the effect; **F** oe- LINC01158 reduced the cells invasive capacity, whereas the increase of levels of miR-711 reversed this effect (*** *P* < 0.001 vs. control; ## *P* < 0.01, ### *P* < 0.001 vs. oe-LINC01158 + miR NC)

The regulatory relationship between LINC01158 and miR-711 was verified. Transfection of overexpressed LINC01158 resulted in an increase in its expression level and a decrease in the level of miR-711; however, transfection of oe-LINC01158 + miR mimic did not affect the level of LINC01158, and the expression of miR-711 was significantly upregulated (*P* < 0.01, Fig. 4A-B). It is suggested that LINC01158 as an upstream gene can regulate the expression of miR-711, but miR-711 could not affect the level of LINC01158. In addition, overexpression of LINC01158 decreased cell proliferation, migration, and invasion (*P* < 0.001). In contrast, the addition of miR-711 mimic reversed this effect (*P* < 0.001, Fig. 4C and F).

## Discussion

The incidence of breast cancer is the highest in the world and BC is one of the leading causes of death among female cancers, posing a serious threat to women’s lives and health [18]. Despite the improved treatment of BC in recent years, the prognosis of BC is still a challenge due to its metastatic and recurrent nature [19]. Therefore, an effective prognostic biomarker is urgently needed to improve the poor prognosis of BC.

Long-stranded non-coding RNAs (lncRNAs) have been reported to be involved in the pathogenesis of many cancers [20, 21], and even exist as oncogenic or tumor suppressor factors in cancer, and are potential prognostic and diagnostic markers in many cancers [22, 23]. Therefore, it is important to investigate the potential function of lncRNAs in BC development and their prognostic value. lncRNA LINC01158 is expressed in cancer [15, 16]. In our study, the expression level of lncRNA LINC01158 was notably downregulated in BC cancer tissues. Furthermore, patients with lower LINC01158 expression had shorter overall survival, and multifactorial Cox regression analysis showed that LINC01158 level was an independent prognostic factor in BC.

In addition, lncRNAs can act as sponges for miRNAs and participate in cancer development and progression by regulating miRNAs [24–26]. Bioinformatics analysis showed that LINC01158 was able to specifically bind miR-711 and regulate its expression level. The level of miR-711 was notably higher in BC tissues compared to normal tissues adjacent to cancer. LINC01158 was negatively correlated with the expression level of miR-711. It has been reported that lncRNAs may be involved in the process of cancer development by regulating miRNA expression to affect pathways such as cell division, growth, and migration [27–29]. For example, LINC01158 functions as an oncogene in gliomas by targeting miR-6734-3p to promote cancer cell growth [14]. when LINC01158 was overexpressed in cells, reduced miR-711 expression levels were observed and cell proliferation, migration, and invasion activities were inhibited, suggesting that LINC01158 may exist as a cancer suppressor in BC. However, this effect was reversed when miR-711 expression levels were increased. Therefore, it is likely that LINC01158 is involved in the process of BC genesis and development by targeting the expression level of miR-711. However, we acknowledge some limitations of this study: First, the involvement of LINC01158 targeting miR-711 in BC-related pathways needs to be further investigated; Secondly, more experimental models (e.g. in vivo models and mouse models, etc.) are needed to validate our existing experimental results.

In conclusion, the expression level of LINC01158 may help to predict the occurrence of adverse prognostic events in BC. overexpression of LINC01158 helps to reduce the level of miR-711, which in turn reduces cell proliferation, decreases the migration and invasive ability of BC cells, and delays the development of BC.

## Data Availability

The datasets used and/or analysed during the current study are available from the corresponding author on reasonable request.
